# Longitudinal effects of rTMS on neuroplasticity in chronic treatment-resistant depression

**DOI:** 10.1007/s00406-020-01135-w

**Published:** 2020-05-09

**Authors:** Iris Dalhuisen, Eveline Ackermans, Lieke Martens, Peter Mulders, Joey Bartholomeus, Alex de Bruijn, Jan Spijker, Philip van Eijndhoven, Indira Tendolkar

**Affiliations:** 1 Department of Psychiatry, Radboud University Medical Center, Huispost 961, PO Box 9101, 6500 HB Nijmegen, The Netherlands; 2 Pro Persona Mental Health Care, PO Box 7049, 6503 GM Nijmegen, The Netherlands; 3 Fundacion Salud Mental Respaldo, Caya Punta Brabo 17, Oranjestad, Aruba; 4 Department of Psychiatry, Rijnstate Hospital, PO Box 9555, 6800 TA Arnhem, The Netherlands; 5 Radboud University Behavioural Science Institute, PO Box 9104, 6500 HE Nijmegen, The Netherlands; 6 Donders Institute of Brain Cognition and Behavior, Centre for Neuroscience, PO Box 9104, 6500 HE Nijmegen, The Netherlands; 7 Donders Institute for Brain Cognition and Behavior, Centre for Cognitive Neuroimaging, PO Box 9104, 6500 HE Nijmegen, The Netherlands; 8 Department of Psychiatry and Psychotherapy, University Hospital Essen, Virchowstraße 174, 45147 Essen, Germany

**Keywords:** Depression, rTMS, Neuroplasticity, Amygdala, Hippocampus, Cortical thickness, Cingulate gyrus

## Abstract

Major depressive disorder (MDD) is amongst the most prevalent of psychiatric disorders. Unfortunately, a third of patients will not respond to conventional treatments and suffer from treatment-resistant depression (TRD). Repetitive transcranial magnetic stimulation (rTMS) has been proven effective in treating TRD. The research suggests that rTMS acts via neuroplastic effects on the brain, which can be measured by changes in hippocampal and amygdala volume as well as cortical thickness. This sham-controlled study investigates longitudinal effects of rTMS on the volumes of the hippocampus and amygdala and cortical thickness in patients with chronic TRD. 31 patients received 20 sessions of high-frequency rTMS (*N* = 15) or sham treatment (*N* = 16) over the left dorsolateral prefrontal cortex during 4 consecutive weeks. Using structural magnetic resonance imaging, we investigated longitudinal treatment effects on hippocampus and amygdala volume as well as thickness of the paralimbic cortex. We found no clinical differences between the active and sham rTMS group. Longitudinal changes in hippocampal and amygdala volume did not differ significantly, although males showed a significant decrease in left amygdala volume, irrespective of treatment group. Changes in cortical thickness of the paralimbic cortex differed significantly between the active and sham groups. Most notably, the increase in cortical thickness of the isthmus of the left cingulate gyrus was greater in the active as compared to the sham rTMS group. Our data suggest that rTMS can induce neuroplastic changes, particularly in cortical thickness, independent of treatment response. We also found longitudinal changes in amygdala volume in males. For clinical effects to follow these neuroplastic effects, more intensive rTMS treatment might be needed in chronically depressed patients.

Trial registration number: ISRCTN 15535800, registered on 29-06-2017.

**Electronic supplementary material:**

The online version of this article (10.1007/s00406-020-01135-w) contains supplementary material, which is available to authorized users.

## Introduction

Major depressive disorder (MDD) is a disabling psychiatric disorder affecting over 300 million people around the world. It greatly impacts quality of life and has severe economic and societal consequences [1]. Up to 35% of MDD patients do not respond sufficiently to first-line treatments with antidepressant medication or psychotherapy [2]. Patients with treatment-resistant depression (TRD) have a high risk for chronicity and often also suffer from comorbid disorders and suicide attempts, emphasizing the need for more effective treatment options [3, 4]. From a neurobiological perspective, decreased neuroplasticity is thought to be the most important underlying mechanism to explain treatment resistance and chronicity [5, 6]. This is reflected on a macroscopic level by for example decreased hippocampal and amygdala volume in patients with MDD, which was associated with duration of illness [7, 8]. A decrease in hippocampal volume was also observed in patients with recurrent episodes as compared to first-episode patients and controls [9], and post mortem analyses of brains of MDD patients showed decreases in cortical thickness and neuronal density, which correlated with duration of illness [10].

New treatment options such as brain stimulation could be of added value in the treatment of depression because of their effects on neuroplasticity. Repetitive transcranial magnetic stimulation (rTMS) is a form of non-invasive brain stimulation that is increasingly used for the treatment of depression and has been shown to be effective, with most studies focusing on patients with TRD [11, 12]. Treatment with rTMS consists of a coil, placed against the head, that induces a magnetic field in the targeted cortex that modulates neuronal activity. In accordance with the neurotrophic hypothesis of depression and its treatment, a neuroplastic component in the effects of rTMS has been suggested by several studies. In a study of TRD patients, response to rTMS was associated with an increase in left amygdala volume and unchanged hippocampal volumes, while non-response was associated with a decrease in left hippocampal volume [13]. An increase in hippocampal volume on the side of the brain targeted with rTMS has also been found [14]. These findings indicate remote neuroplastic effects of rTMS that are not limited to the targeted stimulation site.

Besides these volumetric changes in subcortical structures, neuroplastic effects of rTMS have also been observed in the cortex. Increased cortical thickness after rTMS treatment has been found in regions of the left rostral and caudal anterior cingulate cortex (ACC), which correlated with clinical response [15, 16]. Moreover, after rTMS treatment in patients with TRD increases in structural grey matter volume were found in the left ACC, left insula, left superior temporal gyrus and right angular gyrus [17]. Since volume is a function of cortical surface area and cortical thickness, these increases could also be the result of an increase in cortical thickness. In line with these findings, the most relevant neuroplastic effects could be expected in the paralimbic cortex [18].

The aim of this work is to further describe longitudinal changes in chronic treatment-resistant depression. In this study, we describe the neuroimaging results in a pre–post design of a randomized controlled trial in which 31 patients with chronic TRD were treated with either 20 sessions of high-frequency stimulation of the left dlPFC or sham. The clinical results of this trial are reported separately, in which it was concluded that this rTMS protocol was not an effective treatment option for chronic TRD [19]. Here we aimed to investigate the rTMS treatment-induced neuroplastic effects in this group of patients with chronic treatment-resistant depression, despite the lack of clinical response. We specifically studied the longitudinal effects of rTMS on hippocampal and amygdala volumes, and thickness of the paralimbic cortex, using structural magnetic resonance imaging (sMRI). As a secondary question, we wanted to see if these possible volumetric changes or changes in cortical thickness are related to treatment response, as measured by Hamilton Depression Rating Scale (HDRS) [20]. Thirdly, we were interested in whether the degree of treatment resistance is related to neuroplastic changes in the brain.

## Materials and methods

### Participants

Eligible participants were patients with a diagnosis of unipolar MDD without psychotic features, with a chronic course during the last two years and treatment resistance, defined as inadequate response to at least two adequate trials of antidepressants and one trial of psychotherapy. Exclusion criteria for participation included the presence of a current or past relevant somatic or neurological disorder; a comorbid diagnosis of bipolar disorder, schizophrenia or substance dependence disorders; epilepsy; serious head trauma or brain surgery; large or ferromagnetic metal parts in the head (except for a dental wire); implanted cardiac pacemaker or neurostimulator; and pregnancy. Previous treatment with electroconvulsive therapy (ECT) was not considered a reason for exclusion. None of the participants had received ECT within 6 months before entering the trial. The concomitant use of antidepressants and psychotherapy was allowed, as to not endanger the safety of the participant due to aggravation of depressive symptoms. Detailed information regarding the previous and current treatments are presented in Supplementary Tables 1 and 2.

### Study overview

The study was approved by the local ethics committee (CMO region Arnhem–Nijmegen, The Netherlands). Participants were recruited through the outpatient clinics of the department of psychiatry of Radboud University Medical Centre and Pro Persona Mental Health Care. All participants gave written consent prior to participation. The randomized controlled trial was registered in the ISRCTN registry (ISRCTN 15535800). The purpose of the current study is to report a secondary outcome of this trial, namely investigating potential brain changes. The main results will be reported elsewhere.

Patients were randomized to receive either active or sham rTMS treatment. Treatment consisted of 20 sessions in a period of four weeks. Within one week before and within one week after treatment, an MRI-scan was performed, as well as a baseline and post-treatment clinical assessment. A follow-up clinical assessment was scheduled six months after start of the intervention.

### Evaluation and outcome measures

MDD was diagnosed by administration of the Structured Clinical Interview for DSM-IV (SCID) by a trained psychiatrist (P.v.E). Assessment of comorbid psychiatric disorders was done through the Mini Internal Neuropsychiatric Interview (M.I.N.I.). To assess treatment resistance, the Dutch Method for quantification of Treatment Resistance in Depression (DM-TRD) was used [21]. The 17-item Hamilton Depression Rating Scale (HDRS-17) was used to assess severity of depressive symptoms [20]. Response is defined as a ≥ 50% reduction in score on the HDRS-17, whereas remission is defined as a total score ≤ 7 post-treatment.

### rTMS procedure

rTMS was administered using a Magstim Super Rapid^2^ magnetic stimulator (Magstim Company, Whitland, UK) with a 92-mm figure-of-eight coil. Resting motor threshold (rMT) was determined at the beginning of each treatment week. The rMT was defined as the minimal stimulation intensity evoking a visual observation of thumb or finger movement in ≥ 5 out of 10 trials. Stimulation was set at 110% of the rMT. High-frequency (10 Hz) rTMS was administered five days a week for four consecutive weeks. Participants received 60 trains of 50 stimuli each, with a duration of 5 s and an inter-train interval of 25 s, resulting in a total of 3000 pulses per session. Treatment was applied to the left dlPFC, which was located using electrode position F3 of the 10–20 EEG system [22]. The use of F3 for the localization of the dlPFC has been shown to be equally effective as localization based on neuronavigation [23]. For the sham rTMS group, the same parameters were used, the exception being that the orientation of the coil was tilted 45° away from the cortex.

### MRI data acquisition and cortical reconstruction

High-resolution anatomical images of the whole brain were acquired on a 1.5-T Siemens Sonata whole-body scanner (Siemens, Erlangen, Germany) using a three-dimensional T1-weighted magnetization prepared rapid acquisition gradient echo sequence (MPRAGE) with the following acquisition parameters: T1 850 [ms], TR 2250 [ms], TE 3.68 [ms], flip angle 15 [deg], FoV 256 × 256 × 176 [mm], voxel-size 1.0 × 1.0 × 1.0 [mm]. Due to unavailability of the MRI scanner, three patients did not undergo MRI-scanning and could therefore not be included in our analyses. Two patients only had an MRI-scan before the first rTMS session but not after the last rTMS session. These patients were only included in our analyses concerning pre-treatment volumetric measures.

Scans were analyzed using FreeSurfer software (version 5.3, https://surfer.nmr.mgh.harvard.edu/). FreeSurfer includes skull stripping, B1 bias field correction, gray-white matter segmentation and reconstruction of cortical surface models (gray-white boundary surface and pial surface). In cases where dura was included in the gray matter, a manual correction was applied. The software enables automatic labeling of subcortical structures using a probabilistic algorithm. Initially, each image is a rigid body registered to a probabilistic atlas based on manually-labeled images. Then, the image is morphed to the atlas by a non-linear transform and a Bayesian segmentation procedure is employed. Each voxel in the MRI volume is automatically assigned to a neuro-anatomical label based on probabilistic information estimated from a manually-labeled training set. The labeling procedure is not biased by anatomical variability. The segmentation procedure is based on three types of probabilities to disambiguate labels: the likelihood that a given structure occurs at a specific atlas location; the likelihood of the image intensity given that tissue class; and the probability that a voxel belongs to a given tissue class based on likelihood of the spatial configuration of labels. This automated segmentation and labeling procedure has been shown to be of equal accuracy to manual tracing methods and relatively insensitive to changes in acquisition parameters [24, 25].

A longitudinal pipeline implemented in FreeSurfer was used in which the subjects were their own controls [26]. In the longitudinal pipeline, for every patient a template volume is created from the baseline and post-treatment MRI after rTMS treatment, reducing random variation in the processing procedure and improving robustness and sensitivity of the overall longitudinal analysis. The volumes of the amygdala and hippocampus were corrected for brain size, by dividing these volumes by the estimated total intracranial volume (TIV), obtained from FreeSurfer.

### Statistical analysis

All statistical analyses were performed using SPSS Statistics 22.0 (IBM Corp., Armonk NY, USA) and procedures were 2-tailed with significance set at an alpha-level of 0.05, unless stated otherwise. T-tests and chi-squared tests were used to examine differences on demographic and clinical variables between the active and sham groups. To assess the effect of time (pre/post-rTMS) and treatment group (sham/active) on HDRS score, a repeated measures analysis of covariance (ANCOVA) was performed with age and gender as covariates.

To assess if there was a difference in volume change of amygdala and hippocampus between the active and sham group, a repeated measures ANCOVA was performed with time (pre/post-rTMS) and hemisphere (left/right) as within-subject factors, and treatment group (sham/active) as between-subjects factor. Gender and age at baseline were added as covariates. ANCOVAs were done separately for normalized total amygdala and hippocampal volume change. To assess whether volumetric changes were related to treatment response, Pearson correlation analyses were performed between change in HDRS score and change in hippocampal and amygdala volumes. To assess whether volumetric changes are related to degree of treatment resistance, Pearson correlation analyses were performed between baseline DM-TRD score and change in hippocampal and amygdala volumes. For the hippocampus a 1-tailed design was used based on the earlier findings that show an increase in hippocampal size in effective antidepressant treatment [27–29].

Change in cortical thickness between the active and sham group was assessed using QDEC, FreeSurfer’s graphical interface for analyzing group data. The main effect of the treatment group was estimated for the whole brain (in vertex-wise statistical difference maps) using the FreeSurfer question ‘’Does the average longitudinal cortical thickness symmetrized percent change, accounting for gender, differ between active and sham treatment?’’ and including the nuisance factor age.

On the basis of the previous literature, we a priori hypothesized that we would find changes in cortical thickness in the paralimbic cortex, as a result of rTMS treatment. We therefore report differences as significant below an uncorrected *p* value of 0.001 (two-tailed) and at least 100 vertices, which is considered an appropriate threshold when an a priori hypothesis is present [18, 30].

## Results

### Demographic and clinical characteristics

The demographic and clinical variables of the patients are shown in Table 1. Patients in the active and sham group did not differ in these variables except for current use of antidepressants. The trial was discontinued after 31 patients for futility reasons. HDRS-17 scores did not differ from pre- to post-treatment for the whole group (*F*(1, 27) = 0.547, *p* = 0.466), nor between the active and sham group (*F*(2, 26) = 0.120, *p* = 0.731).

**Table 1:** Demographic and clinical characteristics of patients

	Active (*N* = 15)	Sham (*N* = 16)	Total (*N* = 31)	*P*
Female sex	9 (60%)	13 (81%)	22 (71%)	0.193
Age	47.33 ± 11.49	49.69 ± 11.02	48.55 ± 11.12	0.565
Current AD use	7 (47%)	14 (88%)	21 (68%)	0.015*
Previous ECT	6 (40%)	9 (56%)	15 (48%)	0.366
Duration current episode (months)	54.60 ± 26.19	57.88 ± 54.83	56.29 ± 42.73	0.835
Number of episodes	2.87 ± 1.25	3.50 ± 1.27	3.19 ± 1.28	0.171
Age of onset (years)	28.80 ± 12.07	25.19 ± 10.40	26.94 ± 11.20	0.378
DM-TRD score	18.67 ± 2.37	18.19 ± 2.77	18.42 ± 2.55	0.609
HDRS-17 score pre	24.13 ± 4.29	22.69 ± 3.84	23.39 ± 4.06	0.331
HDRS-17 score post	21.00 ± 5.44	18.56 ± 5.61	19.74 ± 5.57	0.230

Values represent mean ± SD or *N* (%). *indicates a statistically significant result

### Volumetric results

Total hippocampal volume neither differed from pre- to post-treatment for the whole group (*F*(1, 22) = 0.222, *p* = 0.642), nor between treatment groups (*F*(2, 21) = 1.743, *p* = 0.200). This was also the case for total amygdala volume (*F*(1, 22) = 1.705, *p* = 0.205 and *F*(2, 21) = 0.878, *p* = 0.359, respectively). Total amygdala volume from pre- to post-treatment significantly interacted with gender, independent of treatment group (*F*(2, 26) = 4.645, *p* = 0.042). Post hoc analyses with paired-samples t-tests showed no difference in total amygdala volume from pre- to post-treatment in males (*t*(6) = 2.230, *p* = 0.067) and in females (*t*(18) =  − 0.009, *p* = 0.993). Further post hoc analyses of volumes of right and left amygdala separately, for males and females separately, showed a significant decrease in left amygdala volume in males; see also Table 2.

**Table 2:** Normalized amygdala volumes pre- and post-treatment in males and females. Volumes represent percentage of total brain volume

		Pre-treatment (%)	Post-treatment (%)	% Change	*p*
Males	Total amygdala	0.2078 ± 0.0228	0.2015 ± 0.0215	− 3.03	0.067
	Right amygdala	0.1122 ± 0.0134	0.1010 ± 0.0133	− 9.98	0.234
	Left amygdala	0.0956 ± 0.0108	0.0916 ± 0.0104	− 4.18	0.045*
Females	Total amygdala	0.2138 ± 0.0252	0.2138 ± 0.0233	0.00	0.993
	Right amygdala	0.1158 ± 0.0126	0.1160 ± 0.0122	0.17	0.708
	Left amygdala	0.0980 ± 0.0134	0.0978 ± 0.0122	0.20	0.837

Values represent mean ± SD. *indicates a statistically significant result

Change in HDRS-17 score from pre- to post-treatment was not correlated with change in total amygdala volume (*r* =  − 0.007, *p* = 0.973) or change in total hippocampal volume (*r* = 0.165, *p* = 0.421). Change in left amygdala volume also did not correlate with change in HDRS-17 score (*r* =  − 0.027, *p* = 0.898). Baseline DM-TRD score was not correlated with change in total amygdala volume (*r* = 0.368, *p* = 0.064) or change in total hippocampal volume (*r* =  − 0.112, *p* = 0.293).

### Cortical thickness results

**Fig. 1:**
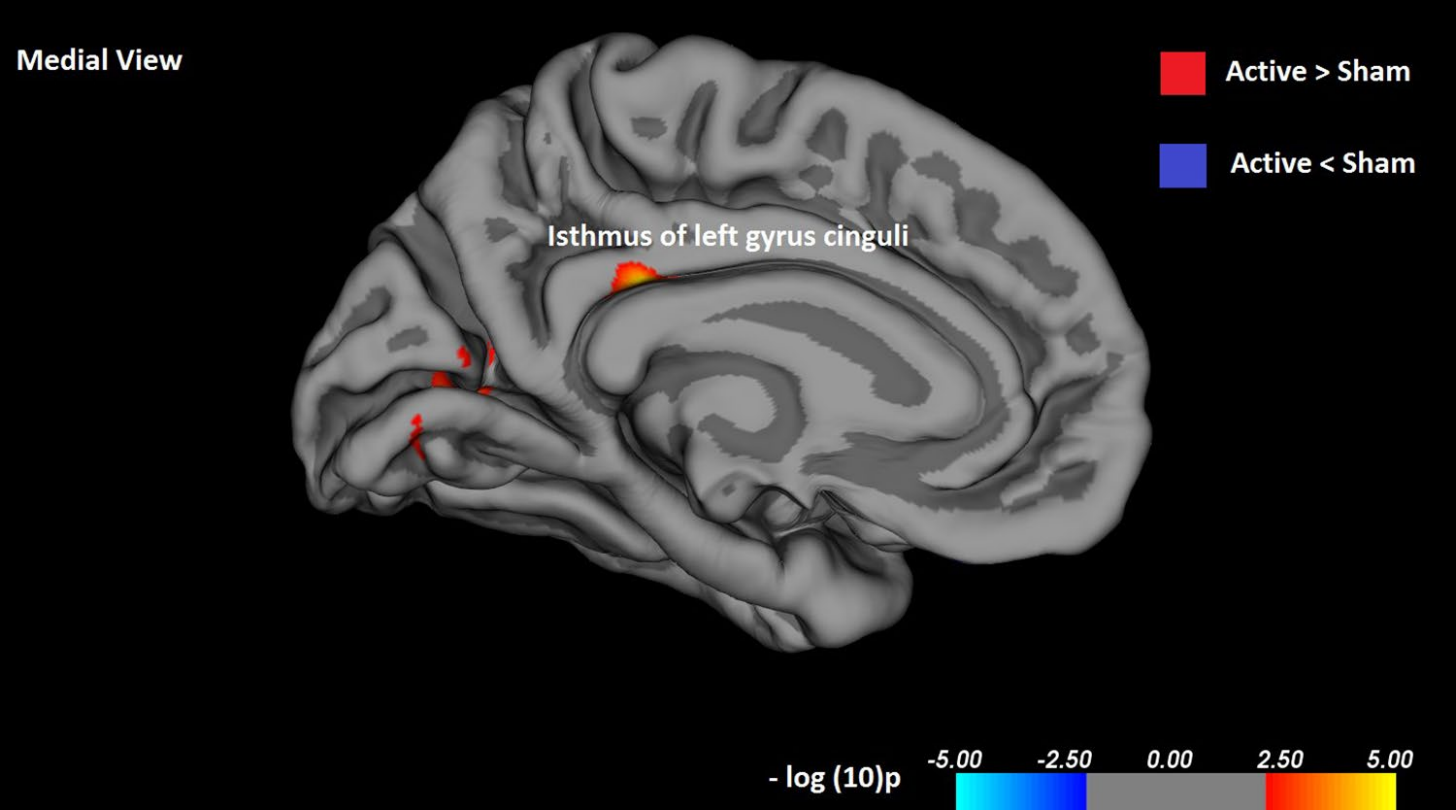
Isthmus of left cingulate gyrus showing significant increased difference in cortical thickness between active and sham rTMS treatment

The vertex-by-vertex analysis showed significant differences between the active rTMS and sham condition in the paralimbic cortex (*p* < 0.001, uncorrected; adjusted for age and gender; see Table3). In the left hemisphere, patients with active rTMS had significantly increased cortical thickness in the left isthmus cingulate gyrus (*p* < 0.000005, see Fig. 1) and the pericalcarine cortex (*p* < 0.0004). In the right hemisphere, this was the case for the post-central gyrus (*p* < 0.0004) and lateral orbitofrontal cortex (*p* < 0.0002). In addition, there were areas of decreased cortical thickness in the left superior parietal lobule (*p* < 0.0007) and in the right superior temporal gyrus (*p* < 0.0007), the post-central gyrus (*p* < 0.0006) and the supramarginal gyrus (*p* < 0.0005).

**Table 3:** Brain regions with significant differences in cortical thickness between active and sham rTMS treatment

Hemisphere	Cortical region	Size (mm^2^)	Coordinates maximum *p* value (mm)a			NVtxs	Uncorrected *p* value
			*x*	*y*	*Z*		
	Active > Sham						
Left	Isthmus cingulate gyrus	123.98	− 6.8	− 33.0	29.7	415	< 0.001**
	Pericalcarine cortex	119.73	− 23.2	− 72.4	5.2	306	< 0.001*
Right	Post-central gyrus	78.79	50.9	− 20.3	53.5	161	< 0.001*
	Lateral orbitofrontal gyrus	45.26	23.9	13.0	− 18.7	119	< 0.001*
	Active < Sham						
Left	Superior parietal lobule	63.45	− 28.0	− 61.7	43.7	171	< 0.001*
Right	Superior temporal lobule	148.28	60.1	− 27.6	1.0	349	< 0.001*
	Post-central gyrus	94.68	59.0	− 10.0	33.2	208	< 0.001*
	Supramarginal gyrus	69.46	50.3	− 37.9	23.6	159	< 0.001*

The results were considered significant if *p* < 0.001 and number of vertices > 100

a. Based on the Talairach coordinates. *indicates a statistically significant result. **indicates a statistically significant results with *p* < 0.0001

## Discussion

To our knowledge, this is the first sham-controlled study that investigated the longitudinal effects of rTMS on hippocampal and amygdala volumes and cortical thickness of the paralimbic cortex in chronic TRD patients. We assessed possible correlations of volumetric changes with clinical treatment response, and whether level of treatment resistance was related to volumetric changes over the course of rTMS treatment. Active and sham rTMS treatment in patients with chronic treatment-resistant major depressive disorder had no clinical effects. Despite the absence of clinical effects, we identified neuroplastic changes in the cingulate cortex, which may indicate that a more intensive treatment protocol could lead to enhanced neuroplastic changes, which in turn could lead to better treatment response.

### Cortical thickness data

Our results show differences in cortical thickness in the active rTMS group over the sham group in patients with chronic TRD. The most pronounced finding to emerge from the analysis is the increased thickness of the left isthmus of cingulate gyrus in the active rTMS group compared to sham. Several studies indicate that cortical thickness is affected in MDD, as regional thinning in the cingulate and orbitofrontal cortex [18, 31, 32]. Neuroimaging studies show that in patients with depression, the structure and connectivity of a subregion of the cingulate cortex, the isthmus, is altered [33–36]. Additionally, stronger functional connectivity between the ACC and the prefrontal cortex has been observed after rTMS treatment, but only in responders [37]. The cingulate cortex is a key area within the fronto-limbic networks involved in emotion, sensory, motor, and cognitive processes [38, 39] and, as part of the paralimbic cortex, is interconnected with the orbitofrontal cortex, amygdala, hippocampus and striatum [38]. The connection between the cingulate cortex and the prefrontal cortex could therefore play an important role in the antidepressant effect of rTMS.

In addition to the increases in cortical thickness, we also identified decreases, specifically in the left superior parietal lobule and in the right superior temporal gyrus, post-central gyrus and supramarginal gyrus. After treatment with rTMS, a decrease in the left subcallosal ACC was found, which did not correlate with clinical improvement [15]. Although the implications are still unclear, it may be that decreases in cortical thickness are also relevant for the neurobiological effects of rTMS treatment in TRD and need to be investigated in future studies.

### Volumetric data

Neither the active rTMS treatment group nor the sham group showed a significant change in hippocampal or amygdala volumes. This was partly consistent with the previous findings of treatment-related volume increase only in the left hippocampus, but no change in amygdala volume [14]. Notably, in this study treatment response was much better than in our study, most likely due to less severe TRD. This suggests that the restricted neuroplastic effects on amygdala and hippocampus volume in our study cannot be simply explained as a function of limited treatment response. Three of the participants in our study were treated with lithium, all of whom were in the sham group. The neuroplasticity-facilitating properties of lithium could have impacted the results; especially because all participants taking lithium were in the same group. Interestingly, we did not find any differences between the active and sham group in volume of the hippocampus and amygdala, the brain regions where the neuroplastic effects of lithium are most pronounced [40], so this is unlikely to have influenced the results. We identified a significant decrease in left amygdala volume in males, independent of treatment group. The results from the previous studies regarding the change in amygdala volume after rTMS treatment have been mixed [13, 14]. Since we did not correct for multiple comparisons, this result needs to be interpreted with care and future studies will need to evaluate this finding.

Furthermore, we found no correlation of volumetric changes with clinical treatment response, which is in line with the previous results [14]. However, a decline in left hippocampal volume specifically in treatment non-responders has also been observed [13]. In line with this, studies on ECT also do not find a relationship between volumetric changes and clinical improvement, irrespective of the large increases in hippocampal volume and strong clinical effects of ECT [41].

Additionally, we examined whether level of treatment resistance correlated with volumetric changes. Our findings do not show evidence for this relationship. A recent review on structural brain characteristics in TRD suggested that volumetric differences in hippocampal volume are likely subtle and therefore current studies lack the statistical power to detect such changes [42], except in large samples [9].

### Study limitations and strengths

This study has several limitations. Firstly, the study had a relatively small sample due to the futility of the trial, which may also limit the statistical power of the neuroimaging results. However, other studies investigating the effects of rTMS on brain structure and volume have used comparable sample sizes whilst also reporting significant findings [13, 14, 16, 17]. In addition, it is an ethical necessity to stop a clinical trial when the risks are found to outweigh the potential benefits [43]. A second limitation is the lack of response to rTMS treatment. rTMS has been shown to be more effective in patients with a lower level of treatment resistance [44], whereas our sample consisted of patients with chronic depression and a very high level of treatment resistance. To illustrate, in a sample of nearly 300 patients with recurrent MDD the average score on the DM-TRD, which can range from 0 to 27, was 9.8 [21]. In our sample, the average scores were 18.7 and 18.2 for the active and sham group, respectively. Since a higher number of pulses per session has been associated with higher response and remission rates [45, 46], rTMS treatment in this study might have been insufficiently intense to result in clinical effect in our highly treatment-resistant chronically depressed sample and an increase in the number of pulses or treatments might increase treatment effect in these patients. Furthermore, participants were not asked to guess their allocated treatment at the end of the study. Expectations regarding a treatment can affect treatment outcome [47]. However, a review regarding blinding integrity showed that subjects are unable to distinguish sham from real rTMS [48]. Moreover, fifteen of the participants had received ECT. Given the neuroplastic effects of ECT, this might have confounded the results [41]. However, when correcting for prior ECT, results remained non-significant. Finally, the use of angulation as a sham method may have resulted in residual brain stimulation, which could have been prevented with the use of a sham coil. However, a true sham procedure is difficult to achieve since each method has its limitations [49].

Nevertheless, by including a sham-controlled rTMS group, we extended on the previous findings since we were able to dissociate whether volumetric changes or changes in cortical thickness are related to the rTMS treatment itself or more global clinical changes during rTMS treatment.

### Future directions

Future research should investigate the neuroplastic effects of rTMS treatment in a larger sample of patients with MDD, ideally in a group with different levels of treatment resistance, to provide more substantial evidence for the influence of rTMS treatment on brain structure and function. For this group of severe treatment-resistant and chronic depression, future TMS studies should use more intensive treatment protocols, by increasing the number of sessions and pulses per session or shift to different TMS protocols with presumed larger neuroplastic effects such as bilateral rTMS or theta-burst stimulation.

## Conclusions

In sum, this is the first sham-controlled study that investigates neuroplastic effects of rTMS treatment on amygdala and hippocampus volume as well as cortical thickness in patients with chronic treatment-resistant depression. We did not find clinical improvement within our sample. However, we did show neurobiological effects of rTMS treatment in the form of changes in cortical thickness in the paralimbic cortex, an area that plays an important role in mood disorders. For clinical effects to follow these neuroplastic effects, more intensive rTMS treatment might be needed in this group of chronically depressed patients. Our results support earlier findings that indicate rTMS-induced neuroplasticity in the cortex. We suggest that rTMS asserts network effects that could potentially be the underlying mechanism responsible for clinical effects. However, findings have so far been mixed and the exact mechanism and meaning of the volumetric changes we found remain unclear and subject for further studies.

## Electronic supplementary material

Below is the link to the electronic supplementary material.

Supplementary file1 (DOCX 14 kb)

Supplementary file2 (DOCX 14 kb)
