# Supplementary material for: Longitudinal effects of rTMS on neuroplasticity in chronic treatment-resistant depression
Source: Eur Arch Psychiatry Clin Neurosci. 2020 May 9;271(1):39–47. doi: 10.1007/s00406-020-01135-w (PMC7867550; doi:10.1007/s00406-020-01135-w)
Supplement: Supplementary file 1 — Supplementary file1 (DOCX 14 kb) [file 406_2020_1135_MOESM1_ESM.docx]

**Supplementary table 1.** Overview of medication during the trial per patient.

| subject - condition | **antidepressant** | **antipsychotic** | **moodstabilizer** | **benzodiazepine** | **other** |
| --- | --- | --- | --- | --- | --- |
| 1 -rTMS | - | - | - | - | - |
| 2 -rTMS | fluoxetine & mirtazepine | - | - | alprazolam & temazepam | promethazine |
| 3 -sham | nortriptyline | - | lithium | lorazepam & clonazepam | - |
| 4 -sham | noritriptyline | - | lithium | - | - |
| 5 -rTMS | - | - | - | lorazepam | melatonin |
| 6 -rTMS | - | quetiapine | - | - | - |
| 7 -sham | fluoxetine | - | - | - | - |
| 8 -sham | noritrytiline | - | - | - | - |
| 9 -rTMS | - | perphenazine | - | temazepam | - |
| 10 -sham | - | - | - | - | melatonine |
| 11 -rTMS | tranylcypromine | - | - | - | melatonine |
| 12 -sham | clomipramine | - | - | lorazepam | - |
| 13 -rTMS | tranylcypromine | - | - | temazepam | - |
| 14 -rTMS | - | - | - | alprazolam | - |
| 15 -sham | - | olanzapine | - | - | - |
| 16 -rTMS | venlafaxine & mirtazapine | quetiapine | - | temazepam & lorazepam | - |
| 17 -sham | tranylcypromine | - | - | lorazepam & temazepam & midazolam | melatonine & levomepromazine |
| 18 -rTMS | amitriptyline | quetiapine | - | flurazepam | methylphenidate |
| 19 -sham | bupropion | quetiapine & haloperidol | - | lorazepam | levomepromazine & promethazine |
| 20 -sham | venlafaxine & trazodon | quetiapine | lithium | - | - |
| 21 -rTMS | tranylcypromine & mirtazapine | haloperidol & olanzapine | - | lorazepam | levomepromazine & promethazine |
| 22 –sham | tranylcypromine | - | - | alprazolam & zoplicon | levomepromazine |
| 23 -rTMS | - | - | - | oxazepam | melatonine |
| 24 -sham | mirtazapine | haldol | - | lorazepam | promethazine |
| 25 -rTMS | clomipramine | aripiprazol | - | temazepam | melatonine |
| 26 -sham | venlafaxine | quetiapine | - | lorazepam | - |
| 27 -rTMS | - | - | - | - | - |
| 28 -sham | sertraline | - | - | - | - |
| 29 -rTMS | - | - | - | oxazepam | levomepromazine |
| 30 -sham | tranylcypromine | - | - | oxazepam | promethazine |
| 31 -sham | venlafaxine | quetiapine | - | - | - |
| total (%) | 21/31 (68%) | 11/31 (35%) | 3/31 (10%) | 19/31 (61%) | 13/31 (42%) |
