# Supplementary material for: Longitudinal effects of rTMS on neuroplasticity in chronic treatment-resistant depression
Source: Eur Arch Psychiatry Clin Neurosci. 2020 May 9;271(1):39–47. doi: 10.1007/s00406-020-01135-w (PMC7867550; doi:10.1007/s00406-020-01135-w)
Supplement: Supplementary file 2 — Supplementary file2 (DOCX 14 kb) [file 406_2020_1135_MOESM2_ESM.docx]

**Supplementary table 2.** Overview of previous treatment in the current episode per patient, based on individual item scores on the DM-TRD.

| subject - condition | **Antidepressant trials** | **Augmentation trials** | **ECT** | **Psychotherapy** | **Intensified treatment** |
| --- | --- | --- | --- | --- | --- |
| 1 -rTMS | 5 | 1 | 1 | 2 | 2 |
| 2 -rTMS | 4 | 1 | 1 | 2 | 2 |
| 3 -sham | 2 | 0 | 0 | 1 | 2 |
| 4 -sham | 5 | 2 | 1 | 1 | 2 |
| 5 -rTMS | 3 | 1 | 0 | 2 | 2 |
| 6 -rTMS | 5 | 1 | 0 | 2 | 1 |
| 7 -sham | 4 | 1 | 0 | 1 | 2 |
| 8 -sham | 3 | 1 | 1 | 2 | 2 |
| 9 -rTMS | 3 | 1 | 0 | 2 | 2 |
| 10 -sham | 3 | 1 | 1 | 1 | 2 |
| 11 -rTMS | 5 | 1 | 0 | 2 | 2 |
| 12 -sham | 2 | 1 | 0 | 1 | 2 |
| 13 -rTMS | 2 | 1 | 0 | 1 | 1 |
| 14 -rTMS | 3 | 1 | 1 | 2 | 2 |
| 15 -sham | 2 | 1 | 1 | 2 | 2 |
| 16 -rTMS | 4 | 2 | 1 | 2 | 2 |
| 17 -sham | 4 | 2 | 1 | 2 | 2 |
| 18 -rTMS | 4 | 1 | 0 | 2 | 2 |
| 19 -sham | 3 | 1 | 1 | 2 | 2 |
| 20 -sham | 4 | 2 | 1 | 2 | 2 |
| 21 -rTMS | 3 | 1 | 1 | 2 | 2 |
| 22 –sham | 3 | 1 | 1 | 1 | 2 |
| 23 -rTMS | 4 | 1 | 0 | 2 | 2 |
| 24 -sham | 2 | 1 | 0 | 0.5 | 2 |
| 25 -rTMS | 3 | 1 | 1 | 2 | 2 |
| 26 -sham | 4 | 2 | 0 | 2 | 2 |
| 27 -rTMS | 2 | 1 | 0 | 2 | 2 |
| 28 -sham | 2 | 0 | 0 | 0.5 | 1 |
| 29 -rTMS | 4 | 2 | 0 | 2 | 2 |
| 30 -sham | 5 | 1 | 1 | 2 | 1 |
| 31 -sham | 2 | 1 | 0 | 2 | 2 |

Antidepressant trials scores: 0 = not used; 1 = 1-2 trials; 2 = 3-4 trials; 3 = 5-6 trials; 4 = 7-10 trials; 5 = >10 trials. Augmentation trials scores: 0 = 0 trials; 1 = 1-2 trials; 2 = 3-4 trials. ECT scores: 0 = no ECT; 1 = ECT (≥8 sessions). Psychotherapy scores: 0.5 = supportive therapy; 1 = empirically supported psychotherapy (≥12 sessions); 2 = 2 or more empirically supported. Intensified treatment scores: 0 = not used; 1 = day treatment (≥12 weeks, ≥3 days/week); 2 = inpatient treatment (≥4 weeks).
